# ‘…but look how many lives would have been saved if this was available then’ Exploring the acceptability of future Human Papillomavirus self‐sampling, and the barriers and facilitators of cervical cancer screening for those with intellectual disabilities in Scotland

**DOI:** 10.1111/bjhp.70092

**Published:** 2026-07-10

**Authors:** Catherine B. White, Lesley M. McGregor

**Affiliations:** ^1^ Division of Psychology University of Stirling Stirling UK

**Keywords:** cervical cancer screening, health inequities, HPV self‐sampling, intellectual disabilities, qualitative research

## Abstract

**Background:**

Cervical cancer screening attendance among women with intellectual disabilities (ID) in Scotland is approximately 30%, significantly below the general population rate of 69% and the WHO's recommended 70% target. HPV self‐sampling could provide a more accessible screening option for this underserved population. This study aimed to understand the views and experiences of women with ID regarding cervical cancer screening and the acceptability of HPV self‐sampling.

**Methods:**

A qualitative study using focus groups was conducted with women with ID who attended third‐sector community groups across Scotland, along with their carers. Four focus groups were held between September and October 2024, with a total of 13 participants (11 women with ID, 2 carers). Data were analysed using reflexive thematic analysis.

**Results:**

Five main themes emerged: (1) National Health Service (NHS) communication as a source of distress; (2) power imbalances between health care providers and women with ID: feeling unheard, dismissed and out of control; (3) enduring consequences of inadequate care; (4) past trauma shapes future avoidance; and (5) self‐sampling as a pathway to empowerment. Participants demonstrated high acceptability of self‐sampling, viewing it as a potential solution to overcome barriers to traditional screening.

**Conclusion:**

This research reveals significant systemic inadequacies in current cervical cancer screening services for women with ID, whilst highlighting self‐sampling's transformative potential. Successful implementation requires comprehensive accessible information, enhanced health care professional training and trauma‐informed care approaches. Self‐sampling could significantly advance health equity goals whilst improving health care outcomes for this underserved population.

**Statement of Contribution**

**What is already known on this subject?**

- Women with intellectual disabilities have significantly lower cervical screening attendance rates compared with the general population.
- Multiple barriers exist including communication difficulties, insufficient appointment time and inadequate professional understanding.
- Human Papillomavirus self‐sampling shows promise for improving screening accessibility in underserved populations.

**What does this study add?**

- First study exploring views of women with ID on HPV self‐sampling in Scotland.
- Identifies specific systemic failures in current screening provision for this population.
- Demonstrates high acceptability of self‐sampling as an empowering alternative to clinic‐based screening.

## INTRODUCTION

Cervical cancer is the fourth most common cancer in women and people with a cervix (WPwC) globally, with an estimated 660,000 new cases identified in 2022 (World Health Organization [WHO], 2024). The WHO's global strategy for cervical cancer elimination prioritizes screening, which has been shown to reduce mortality rates by 41%–92% among attendees across Europe (Jansen et al., 2020). In the United Kingdom, screening is provided free at point of access through the National Health Service, testing for high‐risk Human Papillomavirus (HPV) and cell changes.

High‐risk strains of HPV, a sexually transmitted infection, cause an estimated 99.8% of cervical cancer cases in the United Kingdom (Brown et al., 2018). Scotland utilizes the electronic Scottish Cervical Call Recall System to manage screening invitations, with attendance down by 0.7% to 68.7% in 2021/22 (Public Health Scotland [PHS], 2023). However, significant health inequalities consistently impact engagement with the Scottish Cervical Cancer Screening Programme. As described within the Scottish Government's Equity (2023) in Screening Strategy, social inequalities, influenced by differences in housing, education and employment, drive health inequalities alongside other factors including stigma, discrimination and lack of opportunity. Each of these factors can hinder individuals in making an informed choice around cancer screening and can prevent them from accessing screening. PHS (2022b) indicates that annual disease burdens are predicted to increase by 21% between 2019 and 2043, with two‐thirds of this due to a rise in conditions including cancers (PHS, 2022a).

### Screening attendance in those with intellectual disabilities

A group where cervical cancer screening attendance has consistently been lowest is those with intellectual disabilities (ID). An ID is defined as significant deficits in cognitive, functional and adaptive skills, usually observed where an individual's IQ is <70 (Shogren & Turnbull, 2010). There are an estimated 1.5 million people with ID in the UK (Mencap, 2020).

Recent data from NHS Greater Glasgow and Clyde (2024) found that only 26.5% of eligible individuals with an ID attended for cervical cancer screening, which is significantly lower than both the general population attendance rate of 69% and the WHO's recommended minimum target of 70% for achieving positive mortality outcomes (WHO, 2021). Individuals with ID experience significant inequalities, resulting in a premature mortality gap of approximately 20 years (O'Leary et al., 2018).

Data comparing those with ID to the general population show higher cancer deaths despite often‐lower comparable incidence rates across cancer types (Ward et al., 2024). Research has consistently highlighted lower attendance in cancer screening in this group, which may impact cancer statistics, with cases underdiagnosed and underreported (McCowan et al., 2019).

Multiple barriers to screening attendance have been identified for people with ID, including comprehension difficulties with invitation letters, insufficient appointment duration, physical discomfort, attitudes of professionals and inadequate resource accessibility (Doherty et al., 2020; Gribben & Bell, 2010; Jo's Cervical Cancer Trust, 2019; Watts, 2008). Power et al. (2024) highlighted additional barriers, including insufficient time to prepare for screening and lack of confidence in health care professionals regarding communication with those with ID. Although research remains limited, a comprehensive study of 16,767 individuals in Korea revealed that those with disabilities, including physical, communication, mental (including ID) and cardiopulmonary impairments, were more likely to receive late‐stage cervical cancer diagnoses, receive lower levels of treatment and face higher mortality rates compared to individuals without disabilities (Choi et al., 2021). Findings underscore the importance of developing and adapting screening programmes, offering modalities like self‐sampling to reduce health inequities.

Cervical cancer screening engagement can be understood through several theoretical frameworks. The Theory of Planned Behaviour (TPB; Ajzen, 1991) posits that attendance is shaped by attitudes, subjective norms and perceived behavioural control, with TPB‐based interventions demonstrating efficacy in improving cervical screening participation (Dsouza et al., 2021). The Health Belief Model (HBM; Janz & Becker, 1984) emphasizes how perceptions of susceptibility, severity, benefits and barriers influence motivation to attend; unlike static demographic variables, these perceptions are targetable, highlighting the value of information‐based interventions. For people with ID, lower understanding of screening benefits and greater procedural distress may compound these influences on attendance (Parish et al., 2008). Where novel modalities such as self‐sampling are concerned, the Theoretical Framework of Acceptability (TFA; Sekhon et al., 2017) offers a structured means of evaluating cognitive and emotional responses, encompassing attitudes, anticipated burden, perceived effectiveness and ethicality. Underpinning all of these is health literacy, the ability to access, understand and assess health information to support informed decision‐making (Sørensen et al., 2012). Between one‐third and one‐half of individuals in the European Union demonstrated low health literacy, and this can be substantially lower among people with ID, highlighting implications for both screening attendance and self‐sampling acceptability (Kobayashi et al., 2016).

The Integrated Screening Action Model (I‐SAM; Robb, 2021) and the Determinants of Screening upTake (DOST) model (Dsouza & Van den Broucke, 2025) represent attempts to synthesize existing health behaviour theories into integrated frameworks specifically designed to understand and improve screening participation. The I‐SAM draws on the COM‐B model, the Precaution Adoption Process Model and the Access Framework to propose a staged, multilevel understanding of screening behaviour, positioning capability, opportunity and motivation as key intervention targets across both participant and environmental levels. The DOST model integrates the Health Belief Model, Theory of Planned Behaviour and Theory of Care‐Seeking Behaviour to map psychosocial determinants of screening uptake across individual, interpersonal, sociocultural and health system levels, with particular attention to the belief‐related and affective factors that influence screening decisions in real‐world contexts.

### HPV self‐sampling

HPV screening provides the opportunity for self‐sampling. As of June 2025, the UK NSC recommends that HPV self‐sampling is offered as an option for under‐screened individuals (UK Government, 2025). This option will be available in Scotland, England and Wales from 2026, with this currently in the planning and initial pilot stages (Scottish Parliament, 2025; UK Government, 2025; Welsh Government, 2025). Self‐sampling allows individuals to take the swab themselves at home, offering comparable diagnostic accuracy to clinic‐based samples whilst potentially addressing established barriers to screening attendance (Arbyn et al., 2018; Robb, 2021). By eliminating speculum examination, self‐sampling has demonstrated increased participation among non‐attenders (Chorley et al., 2017; Gok et al., 2021).

YouScreen, a study piloting self‐sampling in London with non‐attenders, demonstrated significant success, particularly with opportunistic delivery (65.5% completion rate), where kits were offered in‐person at the GP practice or community setting upon the patient presenting for a reason which may be unrelated to cervical health (Lim et al., 2024). This enabled the health care professional to provide brief support and encouragement at the point of offer compared with mail delivery (12.9%). Self‐sampling is successfully available in approximately 17 countries, either for everyone or specifically for underserved populations (Serrano et al., 2022). Multiple systematic reviews confirm high acceptability of self‐sampling, particularly regarding convenience and privacy (Kamath & Withers, 2021; Nishimura et al., 2021).

In the United Kingdom, individuals who return a self‐sample and receive an HPV‐positive result are asked to attend their GP practice for a clinic‐based follow‐up appointment (either a repeat HPV test or liquid‐based cytology), as self‐sampling is currently positioned as an accessible entry point into the screening pathway rather than a standalone replacement (NHS North Central London Cancer Alliance, 2021). Results are communicated by letter, with the same recall system used for clinic‐based screening.

Jo's Cervical Cancer Trust (2019) proposed that self‐sampling could enhance accessibility for women with disabilities, specifically emphasizing the need for additional research to determine optimal implementation strategies. A recent systematic review by Power et al. (2024) noted that for the WHO's global strategy of cervical cancer elimination to succeed, elimination must also be achieved for underrepresented groups, including people with ID.

This study represents the first exploration of the direct views and experiences of women with intellectual disabilities in Scotland regarding both cervical cancer screening and HPV self‐sampling. Its significance is underscored by the imminent rollout of self‐sampling across Scotland and the rest of the UK, at a point where we do not have a full understanding of how this initiative will be perceived or experienced by people with ID; a population already facing the most pronounced screening inequalities. Without this understanding, implementation risks replicating the very barriers that have historically excluded this group from equitable screening access.

### Study rationale

Understanding the perspectives of individuals with ID across Scotland regarding self‐sampling is essential, as their engagement and acceptance will be crucial to the initiative's success and to increase the likelihood of health equity. This investigation is particularly timely as the UK approaches potential nationwide implementation of self‐sampling. This research aimed to provide comprehensive insights into barriers to screening attendance and gather perspectives from individuals with ID regarding the self‐sampling kit prior to its implementation.

### Research question

This study was one of two studies completed as part of the researcher's doctoral thesis. This project is publicly available on Open Science Framework: https://osf.io/uzchp/overview?view_only=8096dcc2b68f4a21a5e909f841313403. It addressed the following question: *What are the views of those with an intellectual disability who have a cervix in Scotland and who are eligible for screening, around cervical cancer screening and* *HPV* *self‐sampling, and how can we address their needs prior to a national rollout?*

## METHODOLOGY

### Research design

Focus groups were arranged with women with ID who attended third‐sector community groups. Participants were given the option of attending in‐person or online and could also request a 1:1 interview if preferred. A focus group schedule was developed (see Data S1), incorporating components of the COM‐B model (Capability, Opportunity, Motivation‐Behaviour; Michie et al., 2011), which posits that behaviour results from the interaction of an individual's capability, opportunity and motivation, and the Theoretical Framework of Acceptability (Sekhon et al., 2017), which explores the degree to which an intervention is perceived as appropriate and agreeable by those who encounter it. Both frameworks served as a broad orienting scaffold for focus group question development, ensuring coverage of relevant domains (e.g. capability and opportunity to attend screening, motivation and acceptability of self‐sampling), rather than as a deductive analytical framework. Data were analysed using reflexive thematic analysis (Braun & Clarke, 2019). The Consolidated Criteria for Reporting Qualitative Research (COREQ) informed the reporting structure. Ethical approval was granted in June 2024 by the University of Stirling Ethics Panel.

### Participants and recruitment

The study aimed to recruit 12–15 participants across 3–4 focus groups, with group sizes kept deliberately small (4–6 participants per group) to ensure adequate time and space for supporting communication whilst enabling rich discussion. This approach aligned with recommendations from The National Federation of Voluntary Bodies Providing Services to People with Intellectual Disabilities, who advise that larger groups may lead to difficulties with engagement for this population (Barrett & Kirk, 2000). Participants were also offered the opportunity to invite their carers/supporters to attend as participants; however, carers were not the key focus of this study.

Inclusion criteria are detailed in Table 1. Recruitment occurred through established third‐sector organizations supporting people with ID across Scotland. Organizations were purposively selected to represent diverse geographical areas and demographic characteristics. Initial contact was made with three organizations in April 2024 to gauge interest, with formal recruitment beginning following ethical approval in August 2024. Group leaders presented information sheets to their members to garner interest prior to confirming participant willingness to engage.

**TABLE 1:** Eligibility criteria.

Inclusion criteria	Exclusion criteria
A person with a cervix.	Someone who does not have a cervix.
Aged between 25 and 64 years old (thereby the recommended age for cervical screening).	Aged under 25 years old or over 65 years old.
Have a diagnosed intellectual disability.	Not resident in Scotland.
Have capacity to consent (following the British Psychological Society's Capacity to Consent Checklist).	Unable to participate in a focus group or interview within the project timeline.
Resident of Scotland and is part of an established community group (and their carer, if applicable).	Has a significant or severe impairment which would impact on ability to take part in a focus group or 1:1 interview.
May or may not have attended for a CC screening test.	Does not have capacity to consent (following the British Psychological Society's Capacity to Consent Checklist).
May or may not have, or have had, a CC diagnosis.	
Able to travel to an in‐person focus group (although there will be an alternative option to conduct these over video call/telephone)	

### Measures

**FIGURE 1:**
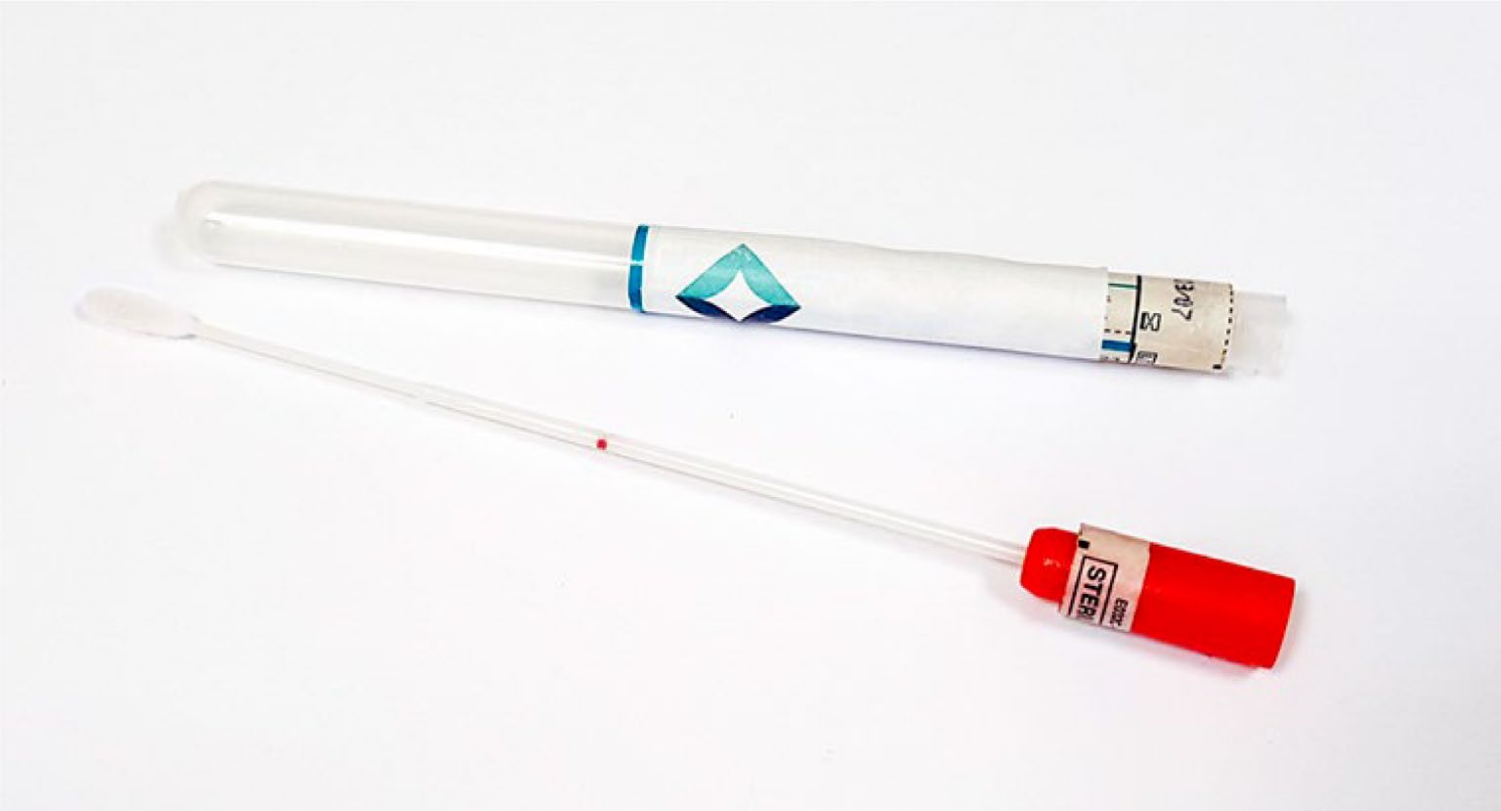
Example of a self‐sampling kit (Royal Australian College of General Practitioners, 2018).

The Focus group schedule was split into three sections: introduction and icebreaker activity; discussion of experiences and attitudes towards cervical cancer screening; and presentation and discussion of the self‐sampling kit (Figure 1). Cards displayed pictures and concise wording for each topic. The schedule included components of both the COM‐B model and the Theoretical Framework of Acceptability. Key information about HPV self‐sampling was provided verbally (Table 2). Data collection occurred during September and October 2024, with groups scheduled to run for 60–90 min.

**TABLE 2:** Key points covered verbally when explaining HPV self‐sampling to participants.

Information provided on HPV self‐sampling
An HPV self‐sampling kit is a test which lets you complete CC screening by yourself at home. It tests for HPV which can cause CC.
There has been a recent pilot of self‐sampling in London, and following this, it might be available as an option in Scotland.
It is likely that self‐sampling will firstly be offered to those who have not gone to their screening appointment following invitation.
You would insert the swab, take the sample and then place this into the test tube. This would then be sent off for testing.
It is not yet known what instructions would be provided with the kit.
It is expected that kits will be posted or given to you at the GP to be completed at home; however, in some countries like Australia, they also provide the option for this to be done in clinic, with support from a nurse or doctor.
It is not yet known how any potential reminder systems would work.

### Stakeholder involvement

Information sheets and consent forms were reviewed by a Senior Clinical Psychologist specializing in ID services, who provided feedback on wording modifications. Documents were shared with group leaders for input to ensure appropriateness. Options to increase accessibility were discussed and made available following stakeholder input, including drawing materials, 1:1 interview options, carer involvement and group leader presence as co‐facilitators. The researcher also spoke with group leaders in advance to understand whether any further accommodations would be helpful; however, no other adjustments were required.

### Consent and confidentiality

Whilst confidentiality was not possible in group settings, rules were discussed emphasizing information not being shared outside the room. Electronic versions of standard and easy‐read information sheets and consent forms were provided to group leaders in advance to discuss with potential participants. Time was allocated at the start of each session to answer questions, check participants understood the documents using teach back (participants repeated what had been said in their own words to confirm understanding) and confirm capacity to consent using the British Psychological Society's Capacity to Consent Checklist. If written consent was not possible (due to physical/visual impairment), verbal consent would have been noted with the affirming signature of a witness; however, this was not needed. The same procedure applied to carers. Where a carer attended as a participant, their consent process and participation in the focus group was conducted entirely separately from the participant with ID. Carers completed their own information sheet and consent form, and their contributions to focus group discussion were treated as independent data in the analysis, distinct from the responses of the participant with ID they supported.

### Procedure

The first two groups occurred in‐person at usual group locations, with the final two groups conducted online via Zoom at participants' request (group 3 received support from the usual group leader and group 4 received support from the husband of the participant with ID). Group leaders had received materials approximately 1 month beforehand and discussed them with potential participants whom they had identified through the eligibility criteria. The researcher assigned the first 10 min for casual conversation to increase comfort levels. Once participants had settled and prior to the main questions, the researcher took time to go over consent forms and ensured these were signed before continuing. For those attending online focus groups, consent forms were provided in advance. Forms were completed by hand and returned to the group leader, who scanned these to the researcher before the groups began. Following consent, recording began using two encrypted audio recorders. Basic demographic information relating to gender was collected. The focus group schedule was followed, with alternative communication methods made available and participants invited to use these. At the end of the group, participants received £5 ‘Love2Shop’ vouchers as thanks. Recordings were uploaded to secure SharePoint folders and professionally transcribed, with transcriptions checked for accuracy and anonymized before analysis.

### Analysis

Reflexive thematic analysis was used, with the researcher critically reflecting on how perspectives and assumptions might influence resulting themes (Braun & Clarke, 2019). Analysis was inductive, exploring themes arising within groups relating to participant views and experiences; neither COM‐B nor TFA were used as deductive coding frameworks. The lead researcher (CW) conducted an initial read‐through of all transcripts to develop familiarization notes before generating initial codes inductively from the data. Quirkos (2023) software organized and facilitated coding. Codes were grouped into candidate themes through an iterative process of sorting and comparison, with the relationship between codes, subthemes and themes documented throughout. Two researchers were involved in the analysis process: CW conducted primary coding and theme development, and LMcG served as an independent check on interpretation. Codes and emerging themes were reviewed in supervision meetings; where differences in interpretation arose, these were resolved through discussion and consensus, with the rationale for decisions recorded in the lead researcher's reflective journal. This process of discussion and revision led to final theme generation. Several strategies were employed to enhance the quality and rigour of the research. The Consolidated Criteria for Reporting Qualitative Research (COREQ) guided reporting standards, ensuring comprehensive documentation of methodology and findings.

The researcher's reflexivity was enhanced through ongoing journaling and supervision discussions, promoting transparency about potential influences on interpretation. Data saturation was assessed through the concept of ‘information power’ (Malterud et al., 2016), recognizing that smaller samples may be sufficient when participants provide rich, detailed accounts relevant to the research question.

### Reflexivity

At the time of this research, the lead researcher (CW) was a Trainee Health Psychologist, who worked full‐time within NHS mental health services as she fulfilled each competency on the professional doctorate. This research was conducted under the supervision of Senior Lecturer and Health Psychologist (LMcG). Conducting the initial stages of this research whilst working in the NHS provided CW with an understanding of the structure and inner workings of the organization and provided access to contacts to support with survey recruitment. Her role also provided experience of working with young people with ID and increased motivation to explore ways to improve accessibility of services for this group. During this project, CW moved into a new role as a Senior Policy Coordinator for an alcohol advocacy charity. This enabled her to consider research from a policy perspective, understanding the work required to enable change, alongside the complexities of decision‐making within government.

CW has a personal interest in this topic due to personal experience of attending CC screening in Scotland. She has a close friend who was diagnosed with CC at a later stage who recognized that fear had prevented her from attending screening earlier. Her perspective as a Trainee Health Psychologist considers that engagement in public health interventions and cancer screening can be understood through behaviours and cognitions, which are influenced by the social determinants of health and other relevant factors. She is aware that imposing one's own beliefs and perceptions on the research process is an inherent aspect of qualitative research; it does not aim to be free from bias but to instead be transparent and accountable. CW engaged in reflexivity throughout the research process to examine her positionality.

## RESULTS

**FIGURE 2:**
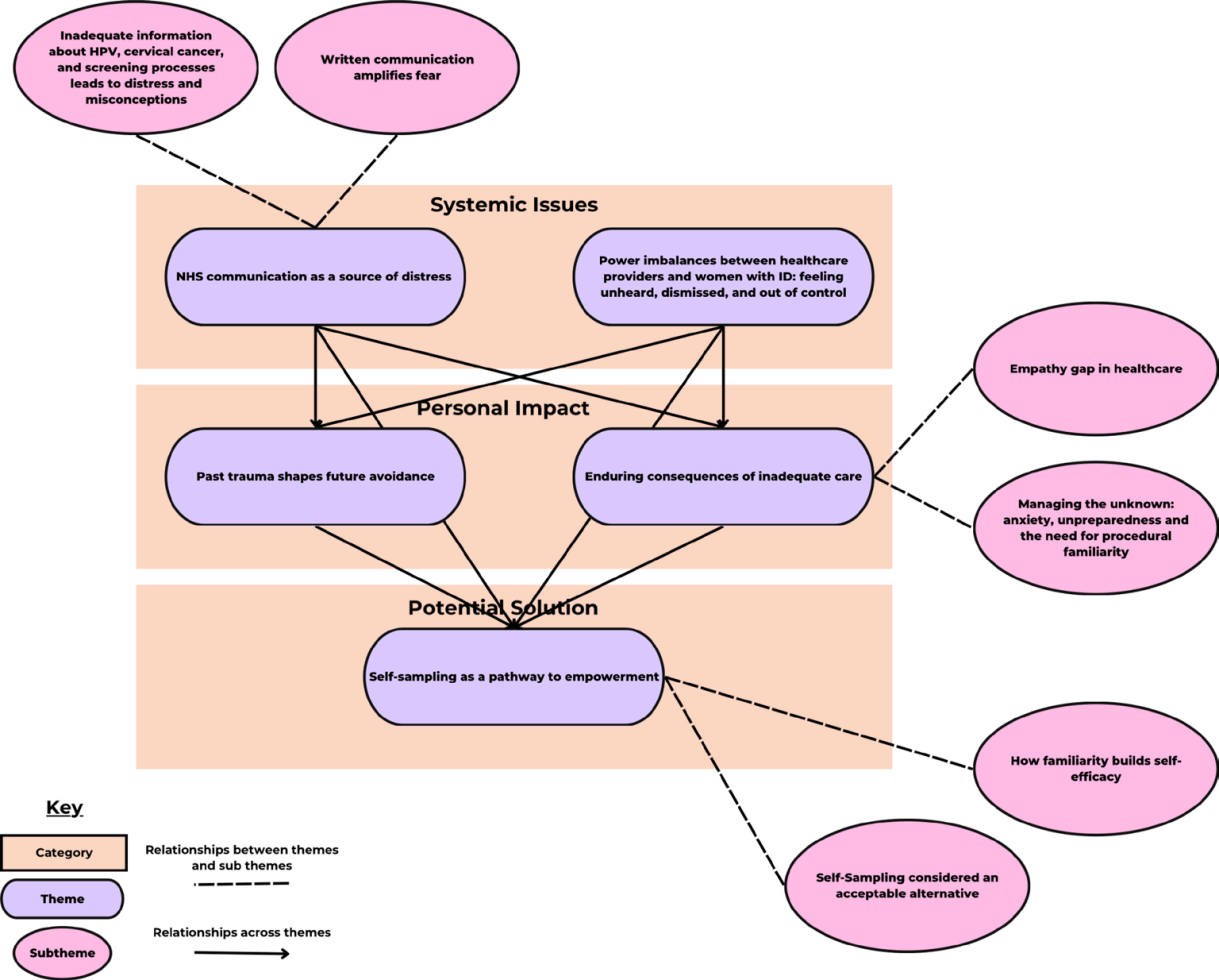
Thematic map of focus group themes and subthemes.

A total of 13 participants took part: 12 females (11 women with ID, 1 carer) and 1 male carer. The majority of participants revealed that they had experience of screening during discussion; however, this was not a requirement. Following analysis, codes were distilled into 5 themes and 6 subthemes, which aligned with 3 categories: systemic issues, personal impact and potential solutions (Figure 2).

### Theme 1: NHS communication as a source of distress

Systemic issues were highlighted across groups. Participants frequently reported inadequate information provision and resulting distress, including feeling insufficiently informed about cervical cancer screening pathway aspects and relevant timescales. Participants shared uncertainty and misunderstandings relating to HPV.

#### Subtheme 1.1

Inadequate information about HPV, cervical cancer and screening processes leads to distress and misconceptions.

Significant distress was reported due to poor levels of accessible information provision. Most participants agreed that waiting for results was the most anxiety‐provoking element of screening, often equating long waits with something being wrong. When no timescale was provided, one participant noted that some women could ‘put themselves into so much of a frenzy…it takes longer for them to be able to come back down from it’.

One participant vividly described the anxiety experienced during the waiting period:

‘…obviously I've got anxiety as well so that doesn't really help…you phone up your doctor like, have you heard anything. No, we've not heard anything, don't worry, it'll come soon, and‚ and it's like, how long's a piece of string…you're sitting there and you're counting the days…’ (FG3)

Participants expressed particular fear around receiving abnormal results via letter without warning or health care professional presence to explain the implications. The lack of preparation for potentially concerning results was described as especially distressing for women with pre‐existing mental health conditions, who found themselves unable to cope with unexpected information delivered through impersonal written communication.

Consensus emerged that communication about the cervical cancer screening programme and its changes was poor. Many reported limited knowledge about HPV and its relationship with cervical cancer, often learning about it only upon diagnosis. This lack of information led to misconceptions, with one participant advising their letter claimed, *‘HPV can be brought on by stress’*. Uncertainty created additional anxiety. Poor communication around HPV and its symptoms was also seen to induce fear in participants, who expressed dichotomous thinking and expectations of the worst‐case scenario.

‘… it is scaring me because at the moment I've had a lot going on down that region of my body…it's, kind of, putting me back to that being back to that basic square one again and I know next year it's coming up and I have to go for this smear test again’ (FG3)

#### Subtheme 1.2: Written communication amplifies fear

Problems with NHS written communication were frequently mentioned. Some shared immediately putting letters ‘in the bucket cos they look scary’, anticipating they would not understand content. NHS letters were described as intimidating and anxiety‐inducing, with one participant noting: ‘when you see a letter from NHS, my heart rate goes like this’ [motions hand quickly on chest].

### Theme 2: Power imbalances between health care providers and women with ID: Feeling unheard, dismissed and out of control

A common theme related to perceived power imbalance leading to loss of control and uninformed consent. Participants shared experiences of stigma and blame from health care professionals, describing feeling dismissed and invalidated, sharing experiences where requests to stop or expressions of pain were ignored.

Women recounted times where, despite clear exclamations of distress and requests to stop, health care professionals continued without acknowledgment: ‘…and he wouldn't listen to me at all…And I'm going, ow, ow, ow, will you please stop, you're hurting me, I can't do anymore. And he wasn't listening at all’ (FG1).

Participants persistently felt dismissed or invalidated, wondering if this was due to their ID, with HCPs believing they were overly sensitive or that their feelings were not as valid. One participant described feeling like, due to her disability, the GP perhaps did not feel that he or she needed to provide additional detail around what was happening and why.

‘…it was like she wasn't listening to me. It was like she'd just thrown me off her list basically, it's not a priority. And then obviously, like, all she was wanting me to do was go and get tests…and I was like, whoa, we're not doing biopsies, we're not doing tests…’ (FG3)

Participants recalled attending for an appointment and being expected to undergo a procedure which they had no prior knowledge of; for some, they were only made aware when lying on the examination table.

‘He said, I'm going to take a biopsy. You never said biopsy, you said examination. So I was really annoyed with that…’ (FG3)

Such descriptions again raised the question around whether informed consent was sought and received. One participant highlighted the consequences of such experiences, where they felt *‘really abused’*. Repeatedly, participants recounted scenarios where they were unaware of what was going to happen to them, including significant surgical procedures.

‘I thought, oh that's just another smear test. Nobody told me, they said, didn't your doctor tell you, I was like, no. So, they removed more than half of my cervix…’ (FG1)

### Theme 3: Enduring consequences of inadequate care

The personal impact of insufficient support was evidenced in this theme. Participants highlighted the critical importance of appropriate accommodations in enabling screening engagement, with most accounts expressing dissatisfaction with experiences to date.

#### Subtheme 3.1: Empathy gap in health care

Participants consistently described hesitation in clinical settings, highlighting how their ID often created uncertainty around what to expect within appointments, yet they struggled to request accommodations: ‘I find people with a learning disability don't know what's in front of them as, do I go to this appointment, do I tell people that I'm scared…’ (FG2).

There was a perceived lack of empathy and understanding from health care professionals who did not seem to recognize when patients needed extra support: *‘And aye respecting that it'll maybe be more distressing for people with disabilities, so being sensitive to that. Be more gentle and stop if they're showing they're not happy’* (FG4).

This highlights the multiple layers of uncertainty faced by women with ID when attending health care appointments, combined with difficulties in self‐advocacy that require health care professionals to be more attuned to non‐verbal cues and more proactive in offering support and reassurance.

#### Subtheme 3.2: Managing the unknown: Anxiety, unpreparedness and the need for procedural familiarity

Anxiety and distress were communicated due to managing the unknown. Participants strongly emphasized the importance of a personalized approach, rejecting ‘one size fits all’ solutions. The opportunity to familiarize themselves with procedures and equipment emerged as a crucial accommodation. Many participants described ‘petrifying’ experiences of encountering unexpected procedures or equipment:

‘I mean, I was petrified when she put the [speculum] thing in and she opened the cervix. And then she puts that tube thing in and I'm like, oh where's that going…’ (FG1)

Whilst a small number of participants described positive experiences where health care professionals took time to walk through procedures and explain equipment, these were exceptional rather than routine. More commonly, women described ‘shock’ when equipment was produced during screening appointments without prior explanation or preparation.

Even when some information was provided, it was frequently inadequate to prepare participants for the reality of the examination:

‘I remember the first time I went and got it done, I saw her bringing these things out and I'm like…I says, can I ask where ‐ what you're going to do with that? She says that's what I use. I went, oh right’ (FG3)

The importance of adequate processing time was highlighted by several participants and carers, who noted that individuals with ID often require additional time to understand and process health information before being able to make informed decisions or feel prepared for procedures:

‘It's almost like she needs that processing time. So for some people they can hear information, ask questions, then the whatever can happen for the sake of this, your smear test. But for [name] she needs extra time initially to really understand…’ (Partner, FG4)

This same carer participant emphasized the importance of those with ID having trusted supporters available to help process information and make decisions, suggesting the need for individuals to ‘go away, speak to us or her friends, and then come back for a second time to do…the procedure or…make a decision’.

Several responses around self‐sampling highlighted the importance of ensuring accommodations, recognizing that additional support was needed. They implored that their views be taken into consideration with self‐sampling. Participants highlighted that instructions must meet their needs to increase confidence in capabilities. This included providing easy‐read instructions, pictures and videos; some suggested a ‘*QR code with a video and easy read…cos reading about it is not going to be the same…as watching how to do it’*.

Participants consistently highlighted the need for reminders. Whilst some could rely on family members to support with this, responses highlighted that they would easily forget about the kit: ‘I'm the type of person, that would probably come through the door, and I would probably put it down, and then forget I'd put it down there. And then I'd be cleaning the house…and it would be, like, oh bugger, that came in weeks ago’ (FG2).

Responses indicated that reminders should be part of the self‐sampling process, providing prompts to complete and return the kit.

### Theme 4: Past trauma shapes future avoidance

A recurring barrier to cervical screening engagement was fear and anxiety feelings relating to past experiences, leading to ongoing screening avoidance. Many described traumatic encounters creating enduring distress. One participant explained that due to past experiences involving physical and emotional pain plus feelings of deceit, they felt unable to trust any health care professionals: ‘It was a horrible experience…now next year's obviously going to come up, am I going to this appointment? Well at the moment, no. Because I don't trust anybody anymore now’ (FG3).

Fear of cancer terminology emerged as a specific barrier that particularly impacted screening attendance. Several participants noted that past experiences of hearing the word ‘cancer’ from health care professionals had created lasting anxiety that prevented them from attending subsequent appointments:

‘Don't mention it. If I've not got it, please don't mention it. I mean, this is why I think a lot of people don't go for their screenings, because they're scared…the time I went in, obviously the woman was really nasty to me…she went, what's made you not, like‚ well you've come in obviously, but what's made you not want to come and I went‚ sick of hearing the word cancer…’ (FG1)

This account demonstrated the complex interaction between fear of disease, fear of health care encounters and the psychological impact of perceived negative attitudes of health care professionals. This participant had interpreted the health care professional's immediate focus on their previous difficulties with attending—and dismissal of the courage it had taken to attend the present appointment—as ‘nasty’. This created additional trauma that further reinforced her reluctance to engage with screening services.

The repeated mention of cancer terminology, combined with health care professionals focusing on why participants had previously missed screening appointments rather than supporting their current attendance, was identified as a key barrier that prevented women from engaging with cervical screening. This created a paradoxical situation where the very services designed to detect and prevent cancer became associated with fear and trauma, ultimately reducing their effectiveness for the populations most in need of support.

### Theme 5: Self‐sampling as a pathway to empowerment

Participants were generally positive about self‐sampling potential as a solution to overcome screening barriers, leading to regained control and reduced vulnerability feelings. Throughout groups, participants emphasized this could be a valuable alternative to clinician‐led screening, particularly for those with ID facing unique challenges.

**FIGURE 3:**
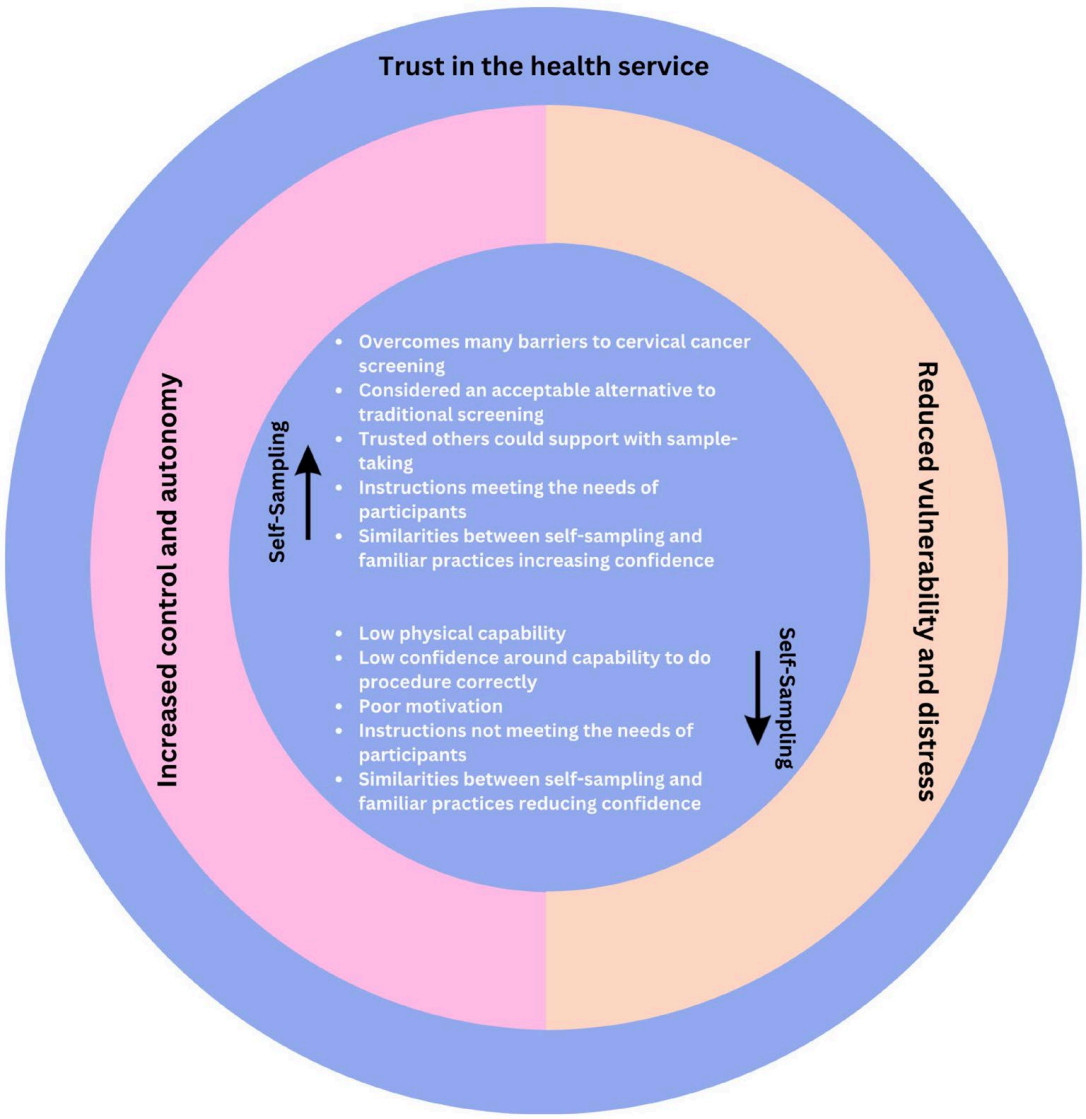
Schematic of key themes influencing engagement with self‐sampling.

Figure 3 summarizes the information shared by participants into a schematic of key factors influencing engagement with self‐sampling. The inner circle highlights the areas that participants felt could both increase engagement and likelihood of success (including high acceptability, trusted others supporting with the process and instructions meeting the needs of the group) and those which could hinder success (including low confidence around capability to do the test correctly and poor motivation). Subtheme 5.1 relates to factors included within this circle, with familiarity increasing the chances of engagement and success of self‐sampling. The inner rings note the two key overarching benefits of self‐sampling for those with ID, which are increased control and autonomy, and reduced vulnerability and distress. Subtheme 5.2 relates to the acceptability of self‐sampling when both of these components are satisfied. The outer ring highlights that trust in the health service ultimately impacts the whole process and whether self‐sampling can be accepted by this group. Theme 5 as a whole encapsulates each component of this schematic, providing insight into how self‐sampling could be a pathway to empowerment.

#### Subtheme 5.1: How familiarity builds self‐efficacy

A recurring thread was the perceived similarity between self‐sampling and other familiar practices including bowel screening, COVID‐19 tests and tampon insertion. For most, this familiarity led to feeling more able to complete HPV self‐sampling: ‘But we had to do things ourselves [during COVID] and we got really used to checking’ (FG3).

For others, however, these comparisons led to them doubting their ability to engage with self‐sampling. Comparisons led to some wondering what additional information would be provided with kits: *‘Not being funny, the COVID tests you had more than that’* (FG2).

Participants recognized that there were additional items provided with other self‐sampling kits including instructions and a return envelope, which were essential in enabling them to complete this.

#### Subtheme 5.2: Self‐sampling considered an acceptable alternative

Most participants were positive about self‐sampling availability. Within each group were members who had previously refused screening attendance, with some strongly against this at group start. By conclusion, however, some had changed minds because of self‐sampling.

Loss of control and power in cervical screening was a major attendance barrier. One of the first things highlighted when discussing self‐sampling was that this gave women power back: ‘…the minute your adrenaline…kicks in, get to that doctor surgery, and they give us these [self‐sampling kits] at home to the women who have been scared, I think it would ease a lot of pressure’ (FG2).

The historical perspective provided by one participant captured the transformative potential of self‐sampling: *‘But*… *look how many lives would have been saved if this was available then’* (FG3).

This powerful statement encapsulated both the hope for future possibilities and the recognition of past missed opportunities. It demonstrated an understanding of screening's life‐saving potential whilst acknowledging how barriers had prevented access to these benefits.

## DISCUSSION

This study presents the perspectives of women with ID on cervical cancer screening and self‐sampling implementation in Scotland. Findings reveal significant systemic barriers within the current cervical cancer screening programme whilst highlighting self‐sampling's potential as a transformative accessibility and engagement tool. This research highlights opportunities to create an inclusive self‐sampling programme at a pivotal time in its launch, where the programme will be more likely to meet the needs of those who should be accessing this, increasing the likelihood of engagement and success.

The three‐category thematic structure including systemic issues, personal impact and potential solutions provides a conceptual framework for understanding how health system determinants interact with individual affective and cognitive responses to produce screening avoidance, and how self‐sampling may interrupt this cycle (Figure 3). Our findings map onto the key domains identified in multilevel determinants of screening uptake models such as the Determinants Of Screening upTake (Dsouza & Van den Broucke, 2025) and the Integrated Screening Action Model (Robb, 2021). Health systems, affective, cognitive and social factors are determinants that do not operate in isolation but interact dynamically. Specifically, three key behavioural pathways can be identified from the data. First, a barrier pathway: Inadequate communication and inaccessible information about HPV and the screening process generate misunderstanding, which heightens fear and anticipatory anxiety, which in turn produces screening avoidance. Second, a trauma pathway: Disempowering or painful clinical experiences, including exclamations of pain being ignored, lack of informed consent and unexpected procedures, erode trust in health care professionals, leading to disengagement from future screening. Third, a self‐sampling facilitation pathway: Offering self‐sampling increases autonomy and reduces the emotional and physical burden associated with clinic‐based procedures, improving acceptability and the likelihood of engagement. Understanding these pathways is essential for designing targeted interventions that address the specific mechanisms underlying screening avoidance in this population, rather than treating the determinants as isolated barriers.

The research demonstrates fundamental inadequacies in the current programme's ability to meet the needs of those with ID, particularly regarding communication, appropriate accommodations and information provision. A key finding was the prevalence of dichotomous thinking patterns among participants, consistent with Cooper et al.'s (2018) observations of cognitive rigidity in individuals with ID. This manifested in participants viewing screening as ‘all bad’ and assessing any symptoms as being ‘back to square one’ whilst being unable to consider the alternative or the potential benefits of screening; a pattern Petrolini et al. (2023) identified as characteristic of ID‐related cognitive processing, which must be considered by HCPs.

The relationship between low health literacy and screening engagement difficulties, previously mentioned by Spadea et al. (2010), appears unchanged over the past decade, suggesting persistent systemic failures in addressing these barriers. This highlights the importance of accessible education around the risks, benefits and process of screening to support informed decision‐making, utilizing frameworks such as the Health Belief Model (HBM; Janz & Becker, 1984). Recent UK data underscore the scale of this challenge: whilst 77.6% of women reported awareness of HPV, only 12.2% demonstrated understanding of the screening procedure itself (Waller et al., 2024). Individuals with ID can experience lower levels of understanding around the advantages of screening, with negative affect and beliefs impacting engagement with screening (Parish et al., 2008), compounding the impact of these knowledge gaps on attendance decisions. Previous education programmes using the HBM have been successful, including Khalil et al.'s (2024) four‐session programme, which led to significant improvements in knowledge and preventative behaviours relating to prostate cancer screening.

A systematic review by Lau et al. (2020) found that, in line with the HBM, the more aware one is about their susceptibility and potential cancer severity, the more likely they are to participate in preventative behaviours such as screening. This must be managed sensitively as participants felt strongly that cancer materials should not be provided unless diagnosed. This aligns with findings from a systematic review from Gil et al. (2024) who identified that those with ID had a ‘deep fear’ around hearing this word, which they suggested could be due to their limited knowledge on the topic. It therefore seems like education is needed, with consideration given on how to approach the topic of cancer in a factual but sensitive manner. Using the HBM to develop education materials which promote awareness of the benefits of early diagnosis and treatment could support to positively adjust individual beliefs around cervical screening. Dsouza et al.'s (2021) application of the TPB to CC screening also provides valuable insight into potential intervention strategies. Their emphasis on fostering positive attitudes and demonstrating procedural accessibility particularly resonates with our findings regarding the importance of accessibility and control in screening decisions.

A key finding concerns inadequacies in current consent practices. Participants' accounts of having pain expressions and requests to stop the procedure ignored align with RCGP (2017) guidance on communication challenges in ID populations. The Learning Disabilities Cancer Strategic Group's (2009) best practice guidelines emphasizing consent importance via behavioural indicators—non‐verbal as well as verbal cues—appear particularly relevant given participants' experiences. Communication strategies around consent should be tailored to the individual; with some participants in the present study sharing that they go non‐verbal when in health care appointments, it is crucial that HCPs take the time to understand individual needs and follow inclusive practices to uphold the rights of their patients.

Perceived Behavioural Control is a key Theory of Planned Behaviour component and crucial factor in whether people feel able to attend cancer screening generally (Lawal et al., 2017) and cervical cancer specifically (Roncancio et al., 2015). Participants justifiably felt vulnerable; whilst initially choosing to attend screening, a lack of understanding and the time taken to build health care professional trust led to feeling without control as procedures progressed.

Evidence from recent systematic reviews confirms this as an area requiring consideration, where health care professionals believed those with ID would not understand cancer screening information, with most directing information towards family members or supporters instead (Power et al., 2024). Mirzaei‐Alavijeh et al.'s (2024) findings on improving screening uptake by increasing perceived control, combined with Kuper et al.'s (2024) emphasis on ‘affirmative, active and accessible’ consent, provide a framework for improving current practices.

Intersectionality, which considers that parts of a person's identity (e.g. gender, sexuality, disability) are not discrete categories but that they instead ‘intersect and reflect social structures of oppression and privilege’ (Kelly et al., 2021), is an important construct to consider. Whilst there are themes that are common across research into cervical cancer screening in women, there are also themes which appear to be unique to the women with ID and which go beyond some of the common difficulties experienced; difficulties around consent procedures and participants not being actively included in decision‐making processes around their health, for example, as well as inadequate consideration of accommodations and non‐verbal indicators of distress. When considered in the context of intersectionality, the themes identified and the disparities experienced by participants are the outcome of interconnected aspects of their identity, power relations and social context. Intersectionality enables us to understand how the participants experience cervical cancer screening differently as a result of their identity and unequal power dynamics. Understanding these differences can enable decision‐makers to develop more effective policy and processes which could support to tackle the structural disadvantages experienced by this group.

The potential of self‐sampling to address current barriers appears significant, with findings indicating high acceptability among participants, including those who had previously declined traditional screening. This aligns with Wedisinghe et al.'s (2022) research showing 97% reengagement intention among defaulters in Dumfries & Galloway when offered self‐sampling. Many systematic reviews have demonstrated high acceptability, with people most often praising convenience, accessibility and elimination of invasive, sometimes traumatizing, physical procedures (Kamath & Withers, 2021; Nishimura et al., 2021). Drawing on the Theoretical Framework of Acceptability (Sekhon et al., 2017), the acceptability of self‐sampling evident in our findings appears to be driven by several distinct dimensions. These include affective attitude (a positive emotional response to the prospect of self‐sampling, contrasting with the negative affect associated with clinic‐based procedures); reduced burden (the elimination of speculum examination and the clinical encounter itself); enhanced self‐efficacy (confidence in the ability to complete the test, mediated by familiarity with analogous practices such as COVID‐19 lateral flow tests and bowel screening); and ethicality (alignment with participants' values around autonomy and bodily control). It is important to note, however, that high acceptability in a qualitative sample does not guarantee uptake at a population level. Structural supports, including clear instructions, reminder systems and access to trusted others during the process, will be essential to translate positive attitudes into completed samples, and future quantitative research should examine whether acceptability predicts actual uptake in this population. Where individuals lack the ability to access, understand and assess health information (Sørensen et al., 2012), their capacity to make truly informed decisions about self‐sampling is constrained, raising important questions about the meaning of consent and autonomous choice. Applying the TFA not only to patients but also to health care professionals, as originally recommended by Sekhon et al., could support decision‐makers to identify and address barriers to implementation at both the individual and system level, improving the conditions under which informed self‐sampling decisions are made.

An important consideration for implementation is that a positive HPV self‐sampling result in the United Kingdom triggers a requirement for a clinic‐based follow‐up appointment (either a repeat HPV test or liquid‐based cytology), as self‐sampling is currently positioned as an accessible entry point into the screening pathway rather than a standalone replacement (NHS North Central London Cancer Alliance, 2021). This creates a potential ‘second hurdle’ for women with ID, where distress may be greater as there is fear around what an invite to clinic means following self‐sampling. Whilst self‐sampling may successfully enable initial HPV testing at home, the barriers identified in this study including power imbalances in clinical environments, risk of inadequate consent procedures, trauma associated with speculum examination and the distress of receiving results without adequate explanation, may be reintroduced at the follow‐up stage. It is therefore essential that the development of self‐sampling programmes for people with ID includes tailored follow‐up support protocols. These should include accessible information available in advance around what a positive result means and what follow‐up involves; the option to attend any follow‐up appointment with a trusted supporter; access to a named health care professional who can provide accessible results communication; and extended appointment time to enable adequate preparation. Self‐sampling will only fully realize its potential for this population if the pathway beyond the initial test is equivalently adapted to their needs; however, the benefit remains that fewer people will need to go through this follow‐up clinic‐based test with self‐sampling.

The potential impact of successful implementation appears substantial. It is believed that one round of self‐sampling when aged 40 in those who have defaulted in Australia could result in 922 fewer cancer diagnoses and 426 fewer deaths from cancer by 84 years of age (Smith et al., 2016). This gains additional significance when considered alongside recent research by Ward et al. (2024) and Herweijer et al. (2023) showing higher cancer mortality rates among women with disabilities.

### Methodological considerations

#### Strengths

Stakeholder engagement was valuable, with participant‐facing documents shared with clinicians experienced in working with and creating documents for people with ID. Data collection via focus groups in different locations provided representative sampling, with group bases ranging from 10% most deprived to 40% least deprived areas of Scotland. Focus groups were considered effective for understanding individuals with ID experiences, with running these within established groups valuable for increasing comfort.

#### Limitations

Only two carer participants were included; arranging standalone carer focus groups or recruiting carers outside community groups could have been valuable for gathering both group perspectives. Whilst study numbers met suggested participant requirements and provided substantial insight, recruitment challenges meant smaller than optimal sample sizes in some groups. The presence of carers within focus groups may have influenced participant responses where individuals may have been less likely to disclose negative or distressing experiences in the presence of their carer, particularly where those experiences were sensitive or personal. Conversely, carer presence may have provided reassurance and supported communication for some participants. This is an inherent methodological consideration in research with adults with ID who may rely on supporter presence. Additionally, individual‐level demographic data beyond gender were not collected, which limits the granularity of quote attribution and prevents analysis of variation by age or other characteristics. Recruiting participants across a wider variety of rural and urban areas may have offered greater insight. Purposely recruiting for participants with a wider variation of experiences with cervical cancer screening would be valuable in future research.

### Implications for practice

Our research indicates that women with ID in Scotland hold complex, often negative views of current cervical cancer screening services, shaped by failures in communication, power imbalance and trauma; yet, they demonstrate high acceptability of HPV self‐sampling, which they perceive as a meaningful pathway to restored autonomy and reduced vulnerability. As Scotland begins to introduce self‐sampling, these findings provide the first evidence base from this population to directly inform that rollout. Findings highlight that equitable implementation will require accessible information, trauma‐informed clinical practice and adapted follow‐up pathways that do not recreate the very barriers self‐sampling sets out to overcome.

This research revealed significant support for self‐sampling from women with ID in Scotland. Drawing from research findings, several recommendations emerge for successful self‐sampling implementation, including comprehensive accessible information materials available in multiple formats, health care professional training including adequate time and materials to build confidence, and educational materials developed using the HBM focusing on raising awareness of cancer susceptibility. These recommendations can be organized across three levels of intervention. With the information environment, accessible multi‐format materials should be developed (including easy‐read, pictorial and video formats, and QR codes linking to demonstration videos) informed by HBM principles to address cognitive barriers such as low awareness of HPV and cancer susceptibility and to provide information on fertility risk, latency period and re‐infection risk (Zhou et al., 2024). Materials should frame cancer information in a factual, sensitive manner that does not amplify fear. Considering clinician interaction and communication, patient‐centred, trauma‐informed care training for health care professionals is essential, encompassing recognition of non‐verbal distress cues, extended appointment time, advance familiarization with equipment and procedures, and inclusive consent practices that centre the voice of the person with ID (Gultekin et al., 2025). At the delivery and logistical level, reminder systems (including text‐based prompts), opportunistic kit offering and the involvement of trusted peers or supporters should be built into self‐sampling programme design (Lim et al., 2024; Wearn & Shepherd, 2024). Importantly, equivalent adaptations must be applied to the follow‐up pathway for those who receive a positive result, to prevent reintroduction of the barriers this study has identified. Implementation should include clear result communication protocols, text‐based reminder systems where preferred and adequate thought given to improving opportunity to engage with self‐sampling for those with ID. Appointments should be available to discuss and see kits, where patients can ask questions and have processing time prior to agreeing to participate. Intervention mapping using a health promotion model such as PRECEDE‐PROCEED (Green & Kreuter, 2005) could be valuable; this would provide a structured approach to planning, implementation and evaluation utilizing the communities in which self‐sampling is delivered, allowing this to be adjusted to address their specific needs. The COM‐B model (Michie et al., 2011) provides a particularly practical framework for designing self‐sampling materials and delivery. As demonstrated by the YouScreen study, materials can be structured to target capability directly through specific written and pictorial instructions, and opportunity through practical enablers such as pre‐paid return envelopes (Lim et al., 2024). Applying the TFA framework to health care professionals as well as to patients would further support implementation, enabling commissioners and practitioners to identify and resolve barriers to offering self‐sampling equitably and with confidence (Sekhon et al., 2017).

## CONCLUSION

This research advances understanding of how self‐sampling could transform cervical cancer screening accessibility for women with ID in Scotland. The findings suggest that successful implementation, grounded in theoretical frameworks and responsive to user needs, could improve outcomes and health care experiences for this underserved population, allowing earlier diagnosis and access to treatment.

The study evidences significant support for self‐sampling from women with ID in Scotland. Self‐sampling could significantly advance health equity goals whilst improving health care outcomes, providing this population with autonomy and choice whilst reducing feelings of vulnerability associated with traditional screening approaches. However, successful implementation requires comprehensive planning, accessible information provision, enhanced health care professional training and trauma‐informed care approaches that prioritize informed consent and patient control.

## AUTHOR CONTRIBUTIONS

**Catherine B. White:** Conceptualization; investigation; writing – original draft; methodology; validation; writing – review and editing; software; formal analysis; project administration; data curation; resources. **Lesley M. McGregor:** Conceptualization; writing – original draft; methodology; validation; writing – review and editing; formal analysis; supervision.

## CONFLICT OF INTEREST STATEMENT

The authors declare no conflicts of interest.

## ETHICS STATEMENT

Ethical approval was granted in June 2024 by the University Ethics Panel (reference number: NICR 2024 17404 13777; 13.06.24).

## Supporting information


Data S1

## Data Availability

The data that support the findings of this study are available from the corresponding author upon reasonable request.

## References

[bjhp70092-bib-0001] Ajzen, I. (1991). The theory of planned behavior. Organizational Behavior and Human Decision Processes, 50(2), 179–211. 10.1016/0749-5978(91)90020-T

[bjhp70092-bib-0002] Arbyn, M. , Smith, S. B. , Temin, S. , Sultana, F. , & Castle, P. (2018). Detecting cervical precancer and reaching underscreened women by using HPV testing on self samples: Updated meta analyses. British Medical Journal, 363, Article k4823. 10.1136/bmj.k4823 30518635 PMC6278587

[bjhp70092-bib-0003] Barrett, J. , & Kirk, S. (2000). Running focus groups with elderly and disabled elderly participants. Applied Ergonomics, 31(6), 621–629. 10.1016/S0003-6870(00)00031-4 11132046

[bjhp70092-bib-0004] Braun, V. , & Clarke, V. (2019). Reflecting on reflexive thematic analysis. Qualitative Research in Sport, Exercise and Health, 11(4), 589–597. 10.1080/2159676X.2019.1628806

[bjhp70092-bib-0005] Brown, K. F. , Rumgay, H. , Dunlop, C. , Ryan, M. , Quartly, F. , Cox, A. , Deas, A. , Elliss‐Brookes, L. , Gavin, A. , Hounsome, L. , Huws, D. , Ormiston‐Smith, N. , Shelton, J. , White, C. , & Parkin, D. A. (2018). The fraction of cancer attributable to modifiable risk factors in England, Wales, Scotland, Northern Ireland, and the United Kingdom in 2015. British Journal of Cancer, 118, 1130–1141. 10.1038/s41416-018-0029-6 29567982 PMC5931106

[bjhp70092-bib-0006] Choi, J. Y. , Yeob, K. E. , Hong, S. H. , Kim, S. Y. , Jeong, E. H. , Shin, D. W. , Park, J. H. , Kang, G. W. , Kim, H. S. , Park, J. H. , & Kawachi, I. (2021). Disparities in the diagnosis, treatment, and survival rate of cervical cancer among women with and without disabilities. Cancer Control: Journal of the Moffitt Cancer Center, 28, 10732748211055268. 10.1177/10732748211055268 35042390 PMC8771753

[bjhp70092-bib-0007] Chorley, A. J. , Marlow, L. A. V. , Forster, A. S. , Haddrell, J. B. , & Waller, J. (2017). Experiences of cervical screening and barriers to participation in the context of an organised programme: A systematic review and thematic synthesis. Psycho‐Oncology, 26(2), 161–172. 27072589 10.1002/pon.4126 PMC5324630

[bjhp70092-bib-0008] Cooper, K. , Loades, M. E. , & Russell, A. J. (2018). Adapting psychological therapies for autism—Therapist experience, skills and confidence. Research in Autism Spectrum Disorders, 45, 43–50. 10.1016/j.rasd.2017.11.002 30245739 PMC6150418

[bjhp70092-bib-0009] Doherty, A. J. , Atherton, H. , Boland, P. , Hastings, R. , Hives, L. , Hood, K. , James‐Jenkinson, L. , Leavey, R. , Randell, E. , Reed, J. , Taggart, L. , Wilson, N. , & Chauhan, U. (2020). Barriers and facilitators to primary health care for people with intellectual disabilities and/or autism: An integrative review. BJGP Open, 4(3), bjgpopen20X101030. 10.3399/bjgpopen20X101030 PMC7465578 32605913

[bjhp70092-bib-0010] Dsouza, J. P. , & Van den Broucke, S. (2025). DOST: A consolidated health behavior model that maps factors influencing cancer screening uptake. Archives of Public Health, 83(1), 70. 10.1186/s13690-025-01517-3 40098055 PMC11912733

[bjhp70092-bib-0011] Dsouza, J. P. , Van den Broucke, S. , Pattanshetty, S. , & Dhoore, W. (2021). The application of health behaviour theories to promote cervical cancer screening uptake. Public Health Nursing, 38(6), 1039–1079. 10.1111/phn.12944 34231254

[bjhp70092-bib-0012] Gil, N. , Cox, A. , Whitaker, K. L. , & Kerrison, R. S. (2024). Cancer risk‐factor and symptom awareness amongst adults with intellectual disabilities, paid and unpaid carers, and healthcare practitioners: A scoping review. Journal of Intellectual Disability Research, 68(3), 193–211. 10.1111/jir.13110 38057951

[bjhp70092-bib-0013] Gok, M. , van Kemenade, F. J. , Heideman, D. A. , Berkhof, J. , Rozendaal, L. , Spruyt, J. W. , Belien, J. A. M. , Babovic, M. , Snijders, P. J. F. , & Meijer, C. J. L. M. (2021). Experience with high‐risk human papillomavirus testing on vaginal brush‐based self‐samples of non‐attendees of the cervical screening program. International Journal of Cancer, 130(5), 1128–1135. 10.1002/ijc.26128 21484793

[bjhp70092-bib-0014] Green, L. , & Kreuter, M. K. (2005). Health program planning: An education and ecological approach. McGraw Hill.

[bjhp70092-bib-0015] Gribben, K. , & Bell, M. (2010). Improving equality of access to cervical screening. Learning Disability Practice, 13, 14–20. 10.7748/ldp2010.09.13.7.14.c7975

[bjhp70092-bib-0016] Gultekin, L. , Nelson, K. N. , Ammerman, B. A. , O'Shaughnessy, H. , & Kuzma, E. K. (2025). Fostering patient‐centered trauma‐informed care: Insights from a first‐time pelvic examination. The Journal for Nurse Practitioners, 21(9), 105504. 10.1016/j.nurpra.2025.105504

[bjhp70092-bib-0017] Herweijer, E. , Wang, J. , Hu, K. , Valdimarsdóttir, A. , Adami, H. O. , Sparén, P. , Sundstrom, K. , & Fang, F. (2023). Overall and cervical cancer survival in patients with and without mental disorders. Journal of the American Medical Association Network Open, 6(9), 2336213. 10.1001/jamanetworkopen.2023.36213 PMC10542737 37773493

[bjhp70092-bib-0018] Jansen, E. E. L. , Zielonke, N. , Gini, A. , Anttila, A. , Segnan, N. , Vokó, Z. , Ivanus, U. , McKee, M. , de Koning, H. J. , & de Kok, I. M. C. M. (2020). Effect of organised cervical cancer screening on cervical cancer mortality in Europe: A systematic review. European Journal of Cancer, 127, 207–223. 10.1016/j.ejca.2019.12.013 31980322

[bjhp70092-bib-0019] Janz, N. K. , & Becker, M. H. (1984). The health belief model: A decade later. Health Education Quarterly, 11(1), 1–47. 10.1177/109019818401100101 6392204

[bjhp70092-bib-0020] Jo's Cervical Cancer Trust . (2019). Behind the headlines: HPV self‐sampling . Retrieved March 6, 2024, from https://www.jostrust.org.uk/about‐us/news‐and‐blog/blog/behind‐headlines‐hpv‐self‐sampling

[bjhp70092-bib-0021] Kamath, A. , & Withers, M. (2021). Human papilloma virus self‐sampling performance in low‐ and middle‐income countries. BMC Women's Health, 21(1), 12. 10.1186/s12905-020-01158-4 33407355 PMC7789658

[bjhp70092-bib-0022] Kelly, C. , Kasperavicius, D. , Duncan, D. , Etherington, C. , Giangregorio, L. , Presseua, J. , Sibley, K. M. , & Straus, S. (2021). ‘Doing’ or ‘using’ intersectionality? Opportunities and challenges in incorporating intersectionality into knowledge translation theory and practice. International Journal for Equity in Health, 20, 187. 10.1186/s12939-021-01509-z 34419053 PMC8379861

[bjhp70092-bib-0023] Khalil, M. I. M. , Ashour, A. , Shaala, R. S. , Allam, R. M. , Abdelaziz, T. M. , & Mousa, E. F. S. (2024). Effect of health belief model‐based educational intervention on prostate cancer prevention; knowledge, practices, and intentions. BMC Cancer, 24(1), 289. 10.1186/s12885-024-12044-9 38438952 PMC10913411

[bjhp70092-bib-0024] Kobayashi, L. C. , Wardle, J. , Wolf, M. S. , & von Wagner, C. (2016). Aging and functional health literacy: A systematic review and meta‐analysis. The Journals of Gerontology. Series B, Psychological Sciences and Social Sciences, 71(3), 445–457. 10.1093/geronb/gbu161 25504637 PMC4834761

[bjhp70092-bib-0025] Kuper, H. , Andiwijaya, F. R. , Rotenberg, S. , & Yip, J. L. Y. (2024). Principles for service delivery: Best practices for cervical screening for women with disabilities. International Journal of Women's Health, 16, 679–692. 10.2147/IJWH.S428144 PMC11034568 38650833

[bjhp70092-bib-0026] Lau, J. , Lim, T. Z. , Jianlin Wong, G. , & Tan, K. K. (2020). The health belief model and colorectal cancer screening in the general population: A systematic review. Preventive Medicine Reports, 20, Article 101223. 10.1016/j.pmedr.2020.101223 33088680 PMC7567954

[bjhp70092-bib-0027] Lawal, O. , Murphy, F. , Hogg, P. , & Nightingale, J. (2017). Health behavioural theories and their application to women's participation in mammography screening. Journal of Medical Imaging and Radiation Sciences, 48(2), 122–127. 10.1016/j.jmir.2016.12.002 31047359

[bjhp70092-bib-0028] Learning Disabilities Cancer Strategic Group . (2009). Best practice in cervical screening for women with learning disabilities . Retrieved November 14, 2024, from https://www.choiceforum.org/docs/bestprac.pdf

[bjhp70092-bib-0029] Lim, A. W. W. , Deats, K. , Gambell, J. , Lawrence, A. , Lei, J. , Lyons, M. , North, B. , Parmar, D. , Patel, H. , Waller, J. , Warwick, J. , & Sasieni, P. D. (2024). Opportunistic offering of self‐sampling to non attenders within the English cervical screening programme: A pragmatic, multicentre, implementation feasibility trial with randomly allocated cluster intervention start dates (YouScreen). EClinicalMedicine, 73, Article 102672. 10.1016/j.eclinm.2024.102672 39429813 PMC11490653

[bjhp70092-bib-0068] Malterud, K. , Siersma, V. D. , & Guassora, A. D. (2016). Sample size in qualitative interview studies: guided by information power. Qualitative Health Research, 26(13), 1753–1760. 10.1177/1049732315617444 26613970

[bjhp70092-bib-0030] McCowan, C. , McSkimming, P. , Papworth, R. , Kotzur, M. , McConnachie, A. , Macdonald, S. , Wyke, S. , Crighton, E. , Campbell, C. , Weller, D. , Steele, R. J. C. , & Robb, K. A. (2019). Comparing uptake across breast, cervical and bowel screening at an individual level: A retrospective cohort study. British Journal of Cancer, 121, 710–714. 10.1038/s41416-019-0564-9 31481732 PMC6889480

[bjhp70092-bib-0031] Mencap . (2020). How common is learning disability . [Fact sheet]. Retrieved from https://www.mencap.org.uk/learning‐disability‐explained/research‐and‐statistics/how‐common‐learning‐disability

[bjhp70092-bib-0032] Michie, S. , van Stralen, M. M. , & West, R. (2011). The behaviour change wheel: A new method for characterising and designing behaviour change interventions. Implementation Science, 6(42), 42. 10.1186/1748-5908-6-42 21513547 PMC3096582

[bjhp70092-bib-0033] Mirzaei‐Alavijeh, M. , Amini, M. , Moradinazar, M. , Eivazi, M. , & Jalilian, F. (2024). Disparity in cognitive factors related to cancer screening uptake based on the theory of planned behaviour. BMC Cancer, 24(845), 845. 10.1186/s12885-024-12607-w 39014335 PMC11251123

[bjhp70092-bib-0034] NHS Greater Glasgow and Clyde . (2024). NHSGGC is improving access to life‐saving cancer screening . Retrieved from https://live.nhsggc.scot/nhsggc‐is‐improving‐access‐to‐life‐saving‐cancer‐screening/#:~:text=26/07/2024,for%20people%20with%20learning%20disabilities

[bjhp70092-bib-0035] NHS North Central London Cancer Alliance . (2021). YouScreen cervical screening made easier . Retrieved from https://www.nclcanceralliance.nhs.uk/our‐work/primary‐care/gp‐info‐youscreen/#:~:text=Women%20who%20are%20HPV%20positive,prevent%20cervical%20cancer%20from%20developing

[bjhp70092-bib-0036] Nishimura, H. , Yeh, P. T. , Oguntade, H. , Kennedy, C. E. , & Narasimhan, M. (2021). HPV self‐sampling for cervical cancer screening: A systematic review of values and preferences. BMJ Global Health, 6(5), e003743. 10.1136/bmjgh-2020-003743 PMC8137189 34011537

[bjhp70092-bib-0038] O'Leary, L. , Cooper, S. A. , & Hughes‐McCormack, L. (2018). Early death and causes of death of people with intellectual disabilities: A systematic review. Journal of Applied Research in Intellectual Disabilities, 31(3), 325–342. 10.1111/jar.12417 28984406

[bjhp70092-bib-0039] Parish, S. L. , Moss, K. , Richman, E. L. , & Taylor, S. J. (2008). Perspectives on health care of adults with developmental disabilities. Journal of Intellectual and Developmental Disability, 46(6), 411–426. 10.1352/2008.46:411-426 19006428

[bjhp70092-bib-0040] Petrolini, V. , Jorba, M. , & Vicente, A. (2023). What does it take to be rigid? Reflections on the notion of rigidity in autism. Frontiers in Psychiatry, 14, 1072362. 10.3389/fpsyt.2023.1072362 36860504 PMC9969081

[bjhp70092-bib-0041] Power, R. , David, M. , Strnadova, I. , Touyz, L. , Basckin, C. , Loblinzk, J. , Jolly, H. , Kennedy, E. , Ussher, J. , Sweeney, S. , Chang, E. L. , Carter, A. , & Bateson, D. (2024). Cervical screening participation and access facilitators and barriers for people with intellectual disability: A systematic review and meta‐analysis. Frontiers in Psychiatry, 15, 1664–0640. 10.3389/fpsyt.2024.1379497 PMC11310793 39132316

[bjhp70092-bib-0042] Public Health Scotland . (2022a). Cervical screening – HPV testing and change of routine screening Intervals: Frequently asked questions for professionals . [Fact sheet]. Retrieved from https://apps.nhslothian.scot/files/sites/2/changes‐to‐cervical‐screening‐programme‐faq.pdf

[bjhp70092-bib-0043] Public Health Scotland . (2022b). Scottish burden of disease study: Forecasting the future burden of disease: Incorporating the impact of demographic transition over the next 20 years. The Scottish Public Health Observatory. Retrieved from https://www.scotpho.org.uk/media/2178/sbod‐forecasting‐briefing‐englishnovember2022.pdf

[bjhp70092-bib-0044] Public Health Scotland . (2023). *Scottish cervical screening programme statistics: Annual update to 31* March 2022. Retrieved from https://publichealthscotland.scot/publications/scottish‐cervical‐screening‐programme‐statistics/scottish‐cervical‐screening‐programme‐statistics‐annual‐update‐to‐31‐march‐2022/

[bjhp70092-bib-0045] Quirkos . (2023). Quirkos 2.5.3 . [Computer Software]. Retrieved from https://www.quirkos.com

[bjhp70092-bib-0046] Robb, K. A. (2021). The integrated screening action model (I‐SAM): A theory‐based approach to inform intervention development. Preventive Medicine Reports, 23, Article 101427. 10.1016/j.pmedr.2021.101427 34189020 PMC8220376

[bjhp70092-bib-0047] Roncancio, A. M. , Ward, K. K. , Sanchez, I. A. , Cano, M. A. , Byrd, T. L. , Vernon, S. W. , Fernandez Esquer, M. E. , & Fernandez, M. E. (2015). Using the theory of planned behaviour to understand cervical cancer screening amongst Latinas. Health Education & Behavior, 42(5), 621–626. 10.1177/1090198115571364 25712240 PMC4932857

[bjhp70092-bib-0048] Royal College of General Practitioners . (2017). Learning disability toolkit . Retrieved from https://www.rcgp.org.uk/clinical‐and‐research/resources/toolkits/learning‐disability‐toolkit

[bjhp70092-bib-0049] Scottish Government . (2023). Health screening: Equity in screening strategy 2023 to 2026 . Retrieved from https://www.gov.scot/publications/scottish‐equity‐screening‐strategy‐2023‐2026/pages/7/

[bjhp70092-bib-0050] Scottish Parliament . (2025). Question reference: S6W‐38966 [date lodged: 25 June 2025] . Retrieved September 14, 2025, from https://www.parliament.scot/chamber‐and‐committees/questions‐and‐answers/question?ref=S6W‐38966

[bjhp70092-bib-0051] Sekhon, M. , Cartwright, M. , & Francis, J. J. (2017). Acceptability of healthcare interventions: An overview of reviews and development of a theoretical framework. BMC Health Services Research, 17(88), 88. 10.1186/s12913-017-2031-8 28126032 PMC5267473

[bjhp70092-bib-0052] Serrano, B. , Ibáñez, R. , Robles, C. , Peremiquel‐Trillas, P. , de Sanjosé, S. , & Bruni, L. (2022). Worldwide use of HPV self‐sampling for cervical cancer screening. Preventive Medicine, 154, Article 106900. 10.1016/j.ypmed.2021.106900 34861338

[bjhp70092-bib-0053] Shogren, K. A. , & Turnbull, H. R. (2010). Public policy and outcomes for persons with intellectual disability: Extending and expanding the public policy framework of AAIDD's 11th edition of intellectual disability: Definition, classification, and Systems of Support. Intellectual and Developmental Disabilities, 48, 375–386. 10.1352/1934-9556-48.5.375 20973700

[bjhp70092-bib-0054] Smith, M. , Lew, J. B. , Simms, K. , & Canfell, K. (2016). Impact of HPV sample self‐collection for underscreened women in the renewed cervical screening program. The Medical Journal of Australia, 204(5), 194. 10.5694/mja15.00912 26985849

[bjhp70092-bib-0055] Sørensen, K. , Van den Broucke, S. , Fullam, J. , Doyle, G. , Pelikan, J. , Slonska, Z. , Brand, H. , & (HLS‐EU) Consortium Health Literacy Project European . (2012). Health literacy and public health: A systematic review and integration of definitions and models. BMC Public Health, 12, 80. 10.1186/1471-2458-12-80 22276600 PMC3292515

[bjhp70092-bib-0056] Spadea, T. , Bellini, S. , Kunst, A. , Stirbu, I. , & Costa, G. (2010). The impact of interventions to improve attendance in female cancer screening amongst lower socioeconomic groups: A review. Preventive Medicine, 50(4), 159–164. 10.1016/j.ypmed.2010.01.007 20093138

[bjhp70092-bib-0058] UK Government . (2025). UK NSC recommends HPV self‐sampling option for under‐screened women in cervical screening programme . Retrieved September 12, 2025, from https://nationalscreening.blog.gov.uk/2025/06/24/uk‐nsc‐recommends‐hpv‐self‐sampling‐option‐for‐under‐screened‐women‐in‐cervical‐screening‐programme/

[bjhp70092-bib-0059] Waller, J. , Waite, F. , & Marlow, L. (2024). Awareness and knowledge about HPV and primary HPV screening among women in Great Britain: An online population‐based survey. Journal of Medical Screening, 31(2), 91–98. 10.1177/09691413231205965 37875156 PMC11083738

[bjhp70092-bib-0060] Ward, L. M. , Cooper, S. , Sosenko, F. , Morrison, D. , Fleming, M. , McCowan, C. , Robb, K. , Hanna, C. R. , Hughes‐McCormack, L. , Dunn, K. , Conway, D. , Henderson, A. , Smith, G. , Truesdale, M. , & Cairns, D. (2024). Population‐based cancer incidence and mortality rates and ratios amongst adults with intellectual disabilities in Scotland: A retrospective cohort study with record linkage. BMJ Open, 14(8), e084421. 10.1136/bmjopen-2024-084421 PMC11331995 39142671

[bjhp70092-bib-0061] Watts, S. (2008). Access to cervical screening for women with learning disabilities. British Journal of Nursing, 17(8), 518–525. 10.12968/bjon.2008.17.8.29205 18563025

[bjhp70092-bib-0062] Wearn, A. , & Shepherd, L. (2024). Determinants of routine cervical screening participation in underserved women: A qualitative systematic review. Psychology & Health, 39(2), 145–170. 10.1080/08870446.2022.2050230.2024 35296200

[bjhp70092-bib-0063] Wedisinghe, L. , Sasieni, P. , Currie, H. , & Baxter, G. (2022). The impact of offering multiple cervical screening options to women whose screening was overdue in Dumfries and Galloway, Scotland. Preventive Medicine Reports, 29, 101947. 10.1016/j.pmedr.2022.101947 36161116 PMC9502330

[bjhp70092-bib-0064] Welsh Government . (2025). Written Statement: Self‐sampling in the cervical screening programme in Wales . Retrieved September 14, 2025, from https://www.gov.wales/written‐statement‐self‐sampling‐cervical‐screening‐programme‐wales

[bjhp70092-bib-0065] World Health Organisation . (2021). New recommendations for screening and treatment to prevent cervical cancer . Retrieved from https://www.who.int/news/item/06‐07‐2021‐new‐recommendations‐for‐screening‐and‐treatment‐to‐prevent‐cervical‐cancer

[bjhp70092-bib-0066] World Health Organisation . (2024). Cervical cancer . [Fact sheet]. Retrieved from https://www.who.int/news‐room/fact‐sheets/detail/cervical‐cancer

[bjhp70092-bib-0067] Zhou, M. , Wu, Y. , Wang, D. , & Cheng, F. (2024). Information needs for cancer screening and associated factors of information‐seeking behaviour: A qualitative systematic review. BMC Public Health, 24(1), 3606. 10.1186/s12889-024-21096-2 39736556 PMC11684248

