# Supplementary material for: ‘…but look how many lives would have been saved if this was available then’ Exploring the acceptability of future Human Papillomavirus self‐sampling, and the barriers and facilitators of cervical cancer screening for those with intellectual disabilities in Scotland
Source: Br J Health Psychol. 2026 Jul 10;31(3):e70092. doi: 10.1111/bjhp.70092 (PMC13354882; doi:10.1111/bjhp.70092)
Supplement: Supplementary file 1 — Data S1 [file BJHP-31-0-s001.docx]

**SUPPLEMENTARY MATERIAL 1 - FOCUS GROUP – SCHEDULE**

**Equipment:** Non-encrypted audio recorder, whiteboard/paper, pens, colourful cards for displaying prompts and topic headings, £5 ‘Love2shop’ vouchers for active participants.

**Location**: Existing Group locations [online if requested by participants/group leader]

**Time**: 60-90 minutes (example timings provided below for a group running 9am-10:30am).

**Facilitators**: XXX, Existing Group Leader.

**Participants**: Individuals with an intellectual disability who have a cervix, and their carer(s), where applicable.

**Agenda**

1. Welcome and introductions (8:30am – 9:05am):
   1. The researcher will be available at the venue 30 minutes before the official start time of the groups, to welcome participants and to support participants to get refreshments (tea, coffee, pastries, biscuits).
   2. A social stories format will be used to clearly display the plan for the day and order of events. There will be individual versions of this provided to the existing Group Leader in advance of the day, and available to each participant in the group.
   3. A script will be available for the facilitator to ensure all key points and wording are used to share information and facilitate discussion.
2. One icebreaker (adapted from suggestions for learners with intellectual disabilities by Granata (2014)) (9:05am – 9:15am):
   1. Favourite Things: Give everyone a piece of paper and have them draw their favourite things. Make this as fun and creative as possible. This can be anything including their favourite TV show, colours, person, food, animal, etc. Share with the group and see if they can find others who like the same thing.
   2. If I Were an Animal: Go round the room and ask everyone to share with the group what animal they would choose to be or feel that they’re most like (can throw a ball of wool while each person remains holding onto a piece of the wool as this is passed amongst participants). Can invite them to give clues to allow others to guess, and to share why this is their favourite/most relatable.
3. Overview, purpose of the group and ground rules (9:15am – 9:25am):
   1. Overview of the topic of cervical cancer screening
   2. Purpose of the study
   3. Purpose of the morning’s focus group and activities
   4. Ground rules (Ground rules will be created as a group and written on a whiteboard at the front of the group. The facilitator will ensure that key rules are included around confidentiality, creating a non-judgmental, safe space, ensuring participants try not to speak over each other and that opinions are respected).
4. Topics for the focus group activity (activity supported by visuals and prompts to aid understanding) (9:25am – 10:15am):
   1. Barriers and facilitators to accessing cervical cancer screening (10 minutes),
   2. Presentation of HPV self-sampling kit to receive initial thoughts and opinions (10 minutes),
   3. Barriers and facilitators of HPV self-sampling (15 minutes),
   4. Discussion of the acceptability of HPV self-sampling within this group (15 minutes).
5. Thanks and close (10:15am – 10:30am).
